# Differentially expressed miRNA profiles of serum-derived exosomes in patients with sudden sensorineural hearing loss

**DOI:** 10.3389/fneur.2023.1177988

**Published:** 2023-06-02

**Authors:** Juhong Zhang, Haizhu Ma, Guijun Yang, Jing Ke, Wenfang Sun, Li Yang, Shaojing Kuang, Hai Li, Wei Yuan

**Affiliations:** ^1^Department of Otorhinolaryngology Head and Neck Surgery, Chongqing General Hospital, Chongqing, China; ^2^School of Basic Medicine, Chongqing Medical University, Chongqing, China; ^3^Department of Otorhinolaryngology Head and Neck Surgery, Xuanhan County People's Hospital, Dazhou, Sichuan, China

**Keywords:** sudden sensorineural hearing loss, exosomes, miRNA transcriptome sequencing, miRNA, biomarkers

## Abstract

**Objectives:**

This study aimed to compare the expressed microRNA (miRNA) profiles of serum-derived exosomes of patients with sudden sensorineural hearing loss (SSNHL) and normal hearing controls to identify exosomal miRNAs that may be associated with SSNHL or serve as biomarkers for SSNHL.

**Methods:**

Peripheral venous blood of patients with SSNHL and healthy controls was collected to isolate exosomes. Nanoparticle tracking analysis, transmission electron microscopy, and Western blotting were used to identify the isolated exosomes, after which total RNA was extracted and used for miRNA transcriptome sequencing. Differentially expressed miRNAs (DE-miRNAs) were identified based on the thresholds of *P* < 0.05 and |log_2_fold change| > 1 and subjected to functional analyses. Finally, four exosomal DE-miRNAs, including PC-5p-38556_39, PC-5p-29163_54, PC-5p-31742_49, and hsa-miR-93-3p_R+1, were chosen for validation using quantitative real-time polymerase chain reaction (RT-qPCR).

**Results:**

Exosomes were isolated from serum and identified based on particle size, morphological examination, and expression of exosome-marker proteins. A total of 18 exosomal DE-miRNAs, including three upregulated and 15 downregulated miRNAs, were found in SSNHL cases. Gene ontology (GO) functional annotation analysis revealed that target genes in the top 20 terms were mainly related to “protein binding,” “metal ion binding,” “ATP binding,” and “intracellular signal transduction.” Kyoto Encyclopedia of Genes and Genomes (KEGG) pathway enrichment analysis revealed that these target genes were functionally enriched in the “Ras,” “Hippo,” “cGMP-PKG,” and “AMPK signaling pathways.” The expression levels of PC-5p-38556_39 and PC-5p-29163_54 were significantly downregulated and that of miR-93-3p_R+1 was highly upregulated in SSNHL. Consequently, the consistency rate between sequencing and RT-qPCR was 75% and sequencing results were highly reliable.

**Conclusion:**

This study identified 18 exosomal DE-miRNAs, including PC-5p-38556_39, PC-5p-29163_54, and miR-93-3p, which may be closely related to SSNHL pathogenesis or serve as biomarkers for SSNHL.

## 1. Introduction

Sudden sensorineural hearing loss (SSNHL) has no identifiable cause and is characterized by a sudden hearing loss of ≥30 dB HL for at least three consecutive frequencies within 72 h (1). SSNHL is mostly unilateral but can occur bilaterally or successively. Its overall incidence rate is increasing globally (2), and treatment responses or effects vary greatly among individuals (3). A considerable number of patients with SSNHL have poor treatment responses (4), which can lead to varying degrees of hearing loss and even permanent severe deafness, thereby seriously affecting patients' quality of life and placing a burden on their families and society. Therefore, it is of great clinical significance to explore the underlying pathogenesis of SSNHL to formulate treatment plans and improve prognosis.

The etiology and pathogenesis of SSNHL have not been fully elucidated. A clear cause, such as certain drugs or tumors, was determined in only 10–15% of the patients with SSNHL, during the onset period (5). The onset of SSNHL may be related to infection, circulatory pathogenesis, or autoimmunity. Infections can be caused by bacteria, spirochetes, and other pathogens, of which viral infections are the most common. Vascular obstruction and changes in the biological activity of vascular endothelial cells can cause cochlear circulatory dysfunction, which is considered the main cause of SSNHL (6–8); however, the exact cause of SSNHL remains a controversial topic.

Exosomes are extracellular vesicles (with a diameter of 30–150 nm) wrapped in a lipid bilayer. They are released from most cell types and can mediate intercellular communication via receptor signaling or cargo delivery to recipient cells (9). In 2018, Wong et al. (10) discovered the existence of exosomes in the inner ear and found that exosomes exert a protective effect against cisplatin- and gentamicin-induced ototoxicity, thus suggesting their potential use as biomarkers. Breglio et al. (11) found that exosomes also protect against aminoglycoside-induced hair cell death, and hair-cell-derived exosomes were found in the perilymph of patients with Meniere's disease, conductive/mixed hearing loss, and genetic SNHL (12). Furthermore, mesenchymal stromal/stem cell-derived exosomes alleviate cisplatin-induced ototoxicity (13–15). However, there have been few studies regarding the relationship between exosomes and SSNHL.

MicroRNAs (miRNAs) are endogenous, short, and non-coding RNAs that regulate gene expression through sequence-specific base pairing with the 3′-untranslated regions (3′-UTRs) of target mRNAs. Circulatory miRNAs are secreted by exosomes, microparticles, vesicles, apoptotic bodies, and protein-miRNA complexes, which exist in saliva, blood, plasma, and other bodily fluids (16). Kamal and Shahidan (17) compared exosomal miRNAs to non-exosomal miRNAs and observed that exosomal miRNAs are more stable during the cell cycle and have a greater potential value as biomarkers. A small number of studies have identified differentially expressed miRNAs (DE-miRNAs) in the serum/plasma of patients with SSNHL, and these DE-miRNAs are functionally enriched (18–20). However, these DE-miRNAs are non-exosomal miRNAs, and exosomal DE-miRNAs have not been identified.

In this study, we compared the expression profiles of serum-derived exosomal miRNAs in patients with SSNHL and normal hearing controls to identify exosomal miRNAs that might be associated with SSNHL pathogenesis or serve as biomarkers for SSNHL.

## 2. Materials and methods

### 2.1. Sample collection and ethics review

Based on clinical practice guidelines on sudden hearing loss (update) (1), we included hospitalized patients (18–65 years old), who met the following diagnostic criteria for unilateral SSNHL within 3 weeks of onset: no treatment, no previous trauma or surgery history, and no cranial nerve damage except for cranial nerve VIII. Normal hearing controls were recruited among hospital staff.

Exclusion criteria were as follows: Meniere's disease, herpes zoster infection, noise-induced deafness, exposure to toxic drugs, other internal diseases of known etiology, meningitis, metabolic diseases, vascular diseases, and autoimmune diseases.

According to the selection and exclusion criteria, six patients with SSNHL and six healthy volunteers were included in this study. Written informed consent was provided by each patient who volunteered before sampling. Clinical information concerning the recruited individuals is shown in Table 1 and Supplementary Figures 1, 2. This study was approved by the Medical Ethics Committee of Chongqing General Hospital (approval no. KYS2021-025-01).

**Table 1:** Physiological and biochemical indices of sudden sensorineural hearing loss (SSNHL) patients and healthy individuals.

**Type**	**Number**	**Sex**	**Age**	**Location**	**Complication**	**Pure tone hearing, dBHL**						**CPR (mg/dl)**	**SBP (mmHg)**	**DBP (mmHg)**	**blood glucose (mmol/L)**	**LDL (mmol/L)**	**TG (mmol/L)**	**ApoB (g/L)**	**Aim**
						**250 Hz**	**500 Hz**	**1,000 Hz**	**2,000 Hz**	**4,000 Hz**	**8,000 Hz**								
SSNHL	1	Male	27	Right	Tinnitus, feeling of ear fullness	65	70	95	110	120	100↓	1.7	110	72	4.82	3.13	1.29	0.89	Sequencing
	2	Female	50	Left	Colitis with tinnitus, feeling of ear fullness, and dizziness	60	55	40	55	50	75	3.9	139	90	5.34	2.64	0.68	0.71	
	3	Female	57	Right	Tinnitus, feeling of ear fullness	95	90	95	95	90	80	11.7	120	72	4.19	2.96	0.86	0.72	
	4	Male	19	Left	Tinnitus, feeling of ear fullness	40	35	40	20	15	15	2.12	135	83	4.85	2.25	1.17	0.6	RT-qPCR
	5	Female	54	Right	Hepatitis B with Tinnitus, feeling of ear fullness	5	0	10	35	70	70	1.45	125	74	4.97	3.78	1.22	1.01	
	6	Male	68	Left	Tinnitus, feeling of ear fullness	55	65	70	55	70	85	0.41	109	77	4.99	1.17	1.41	0.46	
Healthy	1	Male	24	Right	/	−5	0	−5	0	5	5	/	/	/	/	/	/	/	Sequencing
				Left		0	0	−5	0	0	0								
	2	Male	24	Right	/	0	0	−5	0	0	0	/	/	/	/	/	/	/	
				Left		0	−5	0	0	−5	0								
	3	Male	26	Right	/	5	5	0	0	5	5	/	/	/	/	/	/	/	
				Left		0	0	5	0	5	0								
	4	Male	35	Right	/	5	5	0	5	5	10	/	/	/	/	/	/	/	RT-qPCR
				Center		5	0	5	5	10	5								
	5	Female	33	Right	/	0	0	−5	0	5	5	/	/	/	/	/	/	/	
				Left		0	0	5	0	−5	0								
	6	Female	33	Righ	/	−5	0	0	0	5	5	/	/	/	/	/	/	/	
				Left		0	0	0	−5	0	0								

CPR, cardiopulmonary resuscitation; SBP, systolic blood pressure; DBP, diastolic blood pressure; LDL, low-density lipoprotein; TG, triglyceride; ApoB, Apolipoprotein B; RT-qPCR, quantitative real-time polymerase chain reaction.

Peripheral venous blood of the six patients with SSNHL and six controls was collected and centrifuged at 1,900 × *g* for 10 min and 13,000 × *g* for 2 min at 4°C. The obtained serum supernatants were stored at −80°C.

### 2.2. Isolation and identification of serum exosomes

Exosomes were isolated from the serum of patients with SSNHL and controls using high-speed centrifugation at 4°C (21). Briefly, the serum samples were thawed on ice and centrifuged at 500 × *g* for 10 min. The supernatant was transferred to a new sterile centrifuge tube and centrifuged initially at 2,000 × *g* for 30 min and then at 10,000 × *g* for 30 min. The supernatant was then filtered using a 0.22 μm sterile filter, added to an ultra-high-speed centrifuge tube, and centrifuged at 120,000 × *g* for 70 min. The sediments (i.e., exosomes) were resuspended in sterile phosphate buffer saline (PBS).

Concentrations of the isolated exosomes were determined using a bicinchoninic acid (BCA) assay kit (Beyotime Biotechnology, Shanghai, China) according to the manufacturer's instructions, and exosomes were identified using nanoparticle tracking analysis (NTA) (22), transmission electron microscopy (TEM) (23), and Western blotting (24). NTA was performed using a ZetaView PMX 110 instrument (Particle Metrix, Meerbusch, Germany) and its corresponding software (ZetaView 8.02.28) to measure exosome mean, median, and mode sizes (indicated as diameters) as well as the sample concentration. TEM was performed using a JEM 1230 transmission electron microscope (JEOL USA Inc., Peabody, MA, USA) at 110 kV, and images were captured with an UltraScan 4000 CCD camera & First Light Digital Camera Controller (Gatan Inc., Pleasanton, CA, USA) to visualize the exosome morphology and ultrastructure. Anti-TSG101 (1:1,000 dilution), anti-CD9 (1:500 dilution), and anti-HSP70 (1:2,000 dilution) were used as primary antibodies and incubated overnight at 4°C. Goat anti-rabbit IgG (H + L)-HRP (1:5,000 dilution) was used as the secondary antibody and incubated at 37°C for 1 h. Then, 1 × PBST was used to wash the membrane for 5 min each time, and chemiluminescent development was monitored after washing the film three times.

### 2.3. Exosomal miRNA sequencing

RNAiso Plus (TAKARA, Japan) was used to extract total RNA from the isolated exosomes, which was sent to Lianchuan Biotechnology (Hangzhou, China) for miRNA sequencing (*n* = 3). TruSeq Small RNA Sample Prep Kits (Illumina, San Diego, USA) were employed for miRNA library preparation and sequencing. The constructed cDNA library products were sequenced using an Illumina Hiseq2500 platform, and the sequence reading was 1 × 50 bp at single ends.

Raw data incorporate sequence and sequencing quality information of Illumina reads in FASTQ format. ACGT101-miR software (v.4.2) was used to perform the following data quality control steps: the removal of 3′ connectors and N sequences to obtain clean data, retention of sequences with the base degree of 18–26 nt, mapping of sequences to Rfam/Repbase databases, and filtering of non-miRNA sequences. The data obtained after quality control, called valid data, were used for subsequent analyses.

### 2.4. Identification of DE-miRNAs and functional analyses

The expression amounts were first normalized to normal values (25), and then DE significance analysis was conducted based on the normal distribution difference algorithm. A differential expression analysis of miRNAs involving SSNHL and normal control groups was performed using DESeq software. DE-miRNAs were identified based on the thresholds of a *p*-value of < 0.05 and |log_2_ fold change (FC)| > 1.

Next, TargetScan (v5.0) (26–28) and miRanda (v3.3a) (29–31) databases were used to predict target genes of the identified DE-miRNAs, and intersections of the two databases were established as the final target genes of the identified DE-miRNAs. The TargetScan algorithm removed target genes whose context score percentile was < 50, and the miRanda algorithm removed target genes whose TargetScan score was ≥50 and miRanda Energy was < -10. Then, predicted genes of the identified DE-miRNAs were submitted for functional analyses, including gene ontology (GO) terms and Kyoto Encyclopedia of Genes and Genomes (KEGG) pathway enrichment analyses.

### 2.5. RT-qPCR

Expression levels of the selected DE-miRNAs were determined using a stem-loop method. Briefly, total exosomal RNA was extracted from the other three exosome samples using RNAiso Plus (TAKARA) according to the manufacturer's instructions. After total RNA extraction, miRNA reverse transcription was performed using the PrimeScript^TM^ II 1st Strand cDNA Synthesis Kit (TAKARA) based on the manufacturer's protocols. Briefly, a 20 μl mixture was prepared using 3 μl RT-Primer (10 μM), 1 μl dNTP Mixture (10 mM each), 300 ng RNA, and RNase-free H_2_O; this mixture was incubated at 65°C for 5 min and 10 μl of it was added to 4 μl 5 × PrimeScript II buffer, 0.5 μl RNase inhibitor (40 U/μl), 1 μl PrimeScript II RTase (200 U/μl), and 4.5μl RNase-free H_2_O. The resulting mixture was first incubated at 42°C for 60 min and then at 95°C for 5 min. Subsequently, the Power SYBR Green PCR Master Mix (Thermo Fisher Scientific, USA) was used for PCR amplification. *U6* served as a reference gene, and the sequences of all primers used are listed in Table 2. The relative expression levels of the selected DE-miRNAs were calculated using the 2^−ΔΔCt^ method.

**Table 2:** Details of PCR primers utilized in this investigation.

**Name of primer**	**Primer sequence (5^′^-3^′^)**
PC-5p-38556_39-Stem-loop	GTCGTATCCAGTGCAGGGTCCGAGGTATTCGCACTGGATACGACGCCGCC
PC-5p-38556_39-Forward	GGAGTTTGGCTGG
PC-5p-29163_54-Stem-loop	GTCGTATCCAGTGCAGGGTCCGAGGTATTCGCACTGGATACGACTCACAC
PC-5p-29163_54-Forward	GCCGGCCGGCGATTTTGATTTTCA
PC-5p-31742_49-Stem-loop	GTCGTATCCAGTGCAGGGTCCGAGGTATTCGCACTGGATACGACAGGCTT
PC-5p-31742_49-Forward	GCGAGAGCGTTCTGT
miR-93-3p_R+1-Stem-loop	GTCGTATCCAGTGCAGGGTCCGAGGTATTCGCACTGGATACGACTCGGGA
miR-93-3p_R+1-Forward	GCGACTGCTGAGCTAGCACT
U6-human	CTCGCTTCGGCAGCACA
U6-h-Reverse	AACGCTTCACGAATTTGCGT
Downstream universal primer sequence	GTGCAGGGTCCGAGGT

### 2.6. Statistical analysis

All experiments were performed with at least three biological replicates, and differences between the two groups of samples were analyzed using Student's *t*-test. Statistical significance was set at a *P*-value of < 0.05.

## 3. Results

### 3.1. Identification of serum-derived exosomes

**Figure 1:**
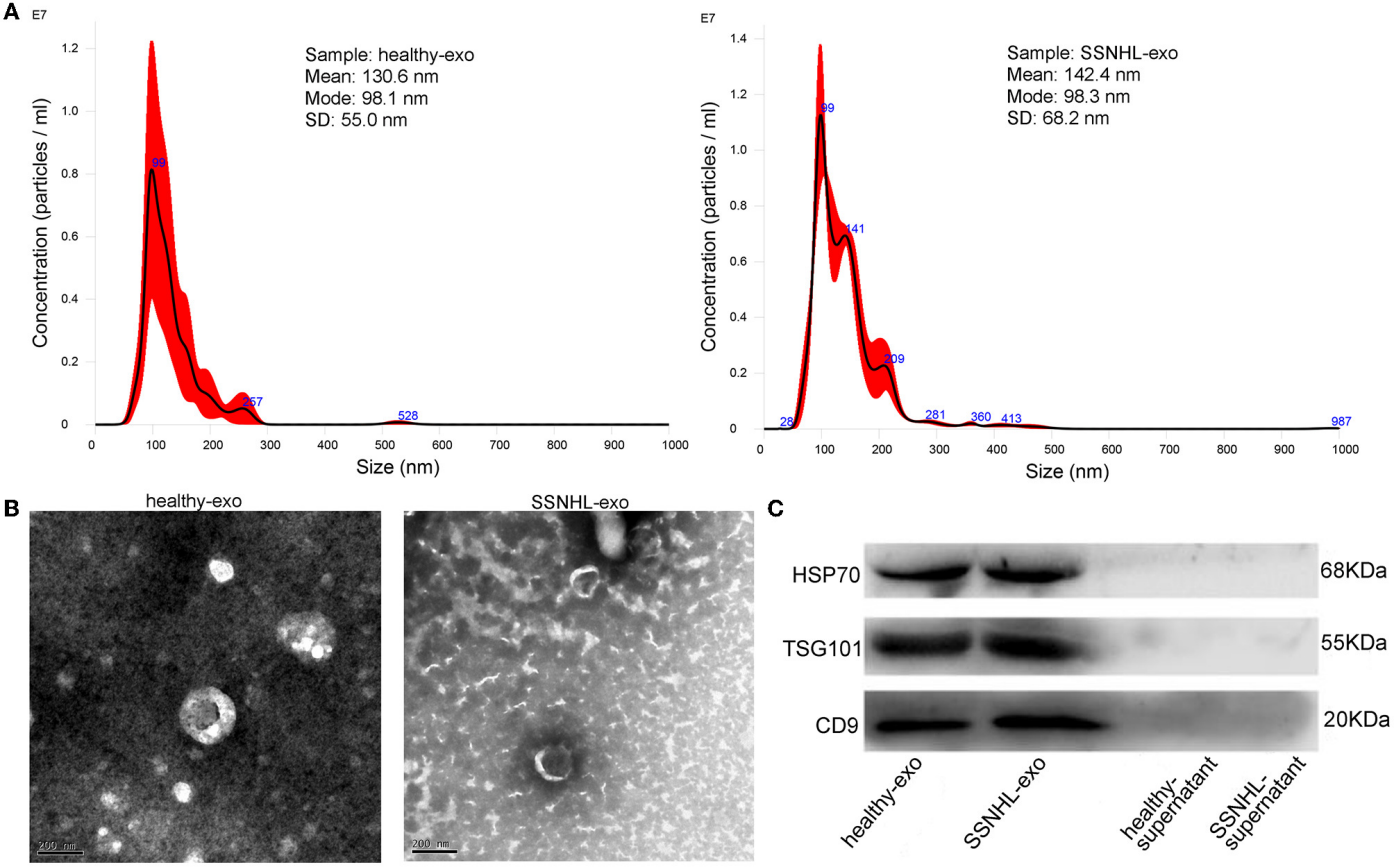
Identification of exosomes isolated from serum of healthy individuals and sudden sensorineural hearing loss (SSNHL) patients. **(A)** Particle size distribution and number of exosomes measured by Nanosight. **(B)** Transmission electron microscopy revealed spherical or saucer-shaped morphology of exosomes with a double-membrane structure. **(C)** Western blotting showed that exosomal surface markers (HSP70, TSG101, and CD9) were all expressed. Healthy-exo, exosomes isolated from healthy individuals; SSNHL-exo, exosomes isolated from SSNHL patients; healthy-supernatant, supernatant from healthy individuals; SSNHL-supernatant, supernatant from SSNHL patients.

NTA revealed that most exosomes were ~30–150 nm in size, and peak sizes were 98.1 and 98.3 nm in healthy control and SSNHL groups, respectively (Figure 1A). TEM showed that the exosomes were spherical or saucer-shaped with a double-membrane structure (Figure 1B). Western blotting showed that exosomal marker proteins HSP70, TSG101, and CD9 were present (Figure 1C). These results indicated that exosomes were successfully isolated from the serum of healthy controls and SSNHL patients.

### 3.2. Quality control of sequence reads and identification of miRNAs

The number of total reads, total bases, and the proportions of each base are shown in Table 3. There were 9,350,833–22,323,603 total reads and 577,118,448–1,138,503,753 total bases. Meanwhile, >98% base call error probability was <1%, and >95% base error probability was <0.1%. The sequencing results were thus considered reliable.

**Table 3:** Quality control and error probability.

**Sample ID**	**Total Reads**	**Total Bases**	**A%**	**T%**	**C%**	**G%**	**N%**	**Q20%**	**Q30%**	**GC%**
C1	9,350,833	476,892,483	23.16	21.77	24.61	30.45	0.01	99.10	97.09	55.06
C2	11,316,048	577,118,448	24.01	21.52	26.17	28.29	0.01	98.82	96.40	54.46
C3	22,323,603	1,138,503,753	22.76	22.86	26.73	27.65	0.00	98.50	95.75	54.37
D1	10,720,432	546,742,032	23.50	21.84	26.50	28.16	0.01	98.68	95.87	54.66
D2	10,186,256	519,499,056	23.50	22.86	25.22	28.41	0.01	98.79	96.20	53.63
D3	11,008,900	561,453,900	22.63	22.36	25.26	29.75	0.01	98.38	95.00	55.01

**Figure 2:**
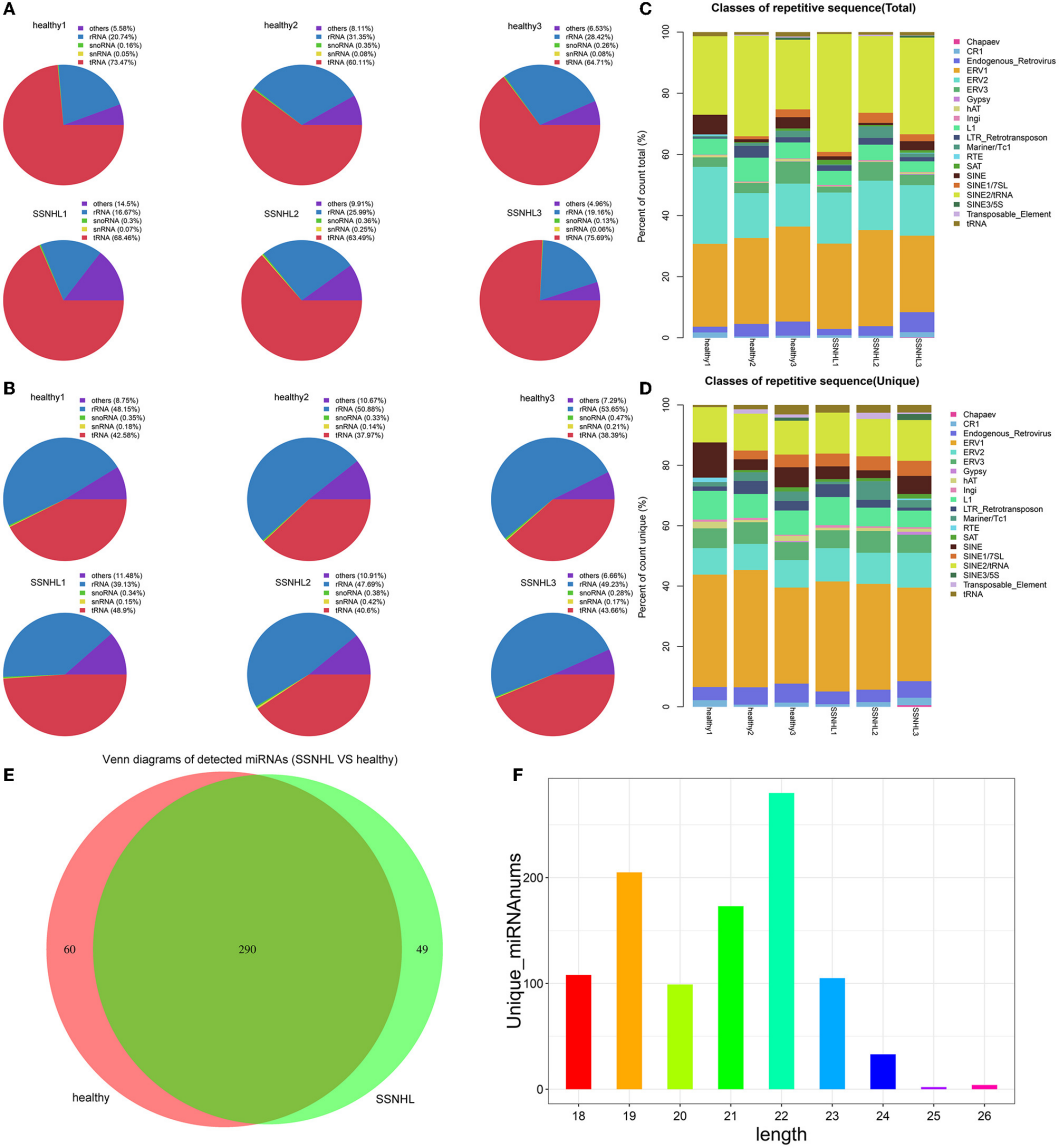
Database comparison and microRNA (miRNA) identification. **(A)** The ratio of rRNA, snoRNA, snRNA, tRNA, and other non-miRNA sequences in total reads to Rfam data. **(B)** The ratio of rRNA, snoRNA, snRNA, tRNA, and other non-miRNA sequences in unique reads to Rfam data. **(C)** The ratio of rRNA, snoRNA, snRNA, tRNA, and other non-miRNA sequences in total reads to Repbase data. **(D)** The ratio of rRNA, snoRNA, snRNA, tRNA, and other non-miRNA sequences in unique reads to Repbase data. **(E)** Venn diagram showing the number of miRNAs detected in the two groups of samples. **(F)** Length distribution of the detected miRNAs.

Rfam and Repbase database alignment analyses were performed to remove non-miRNA and repetitive sequences in the clean data. Total reads and unique reads were counted and visualized as pie and stacked charts, respectively (Figures 2A–D).

The Venn diagram showed that 399 miRNAs were identified in the two groups, including 350 in the control group, 339 in the case group, and 290 that were co-expressed by the two cohorts (Figure 2E). Length distribution analysis indicated that the majority of reads were between 18 and 24 nucleotides (nt) long, with the most common length being 22 nt (Figure 2F).

### 3.3. Screening of DE-miRNAs

**Figure 3:**
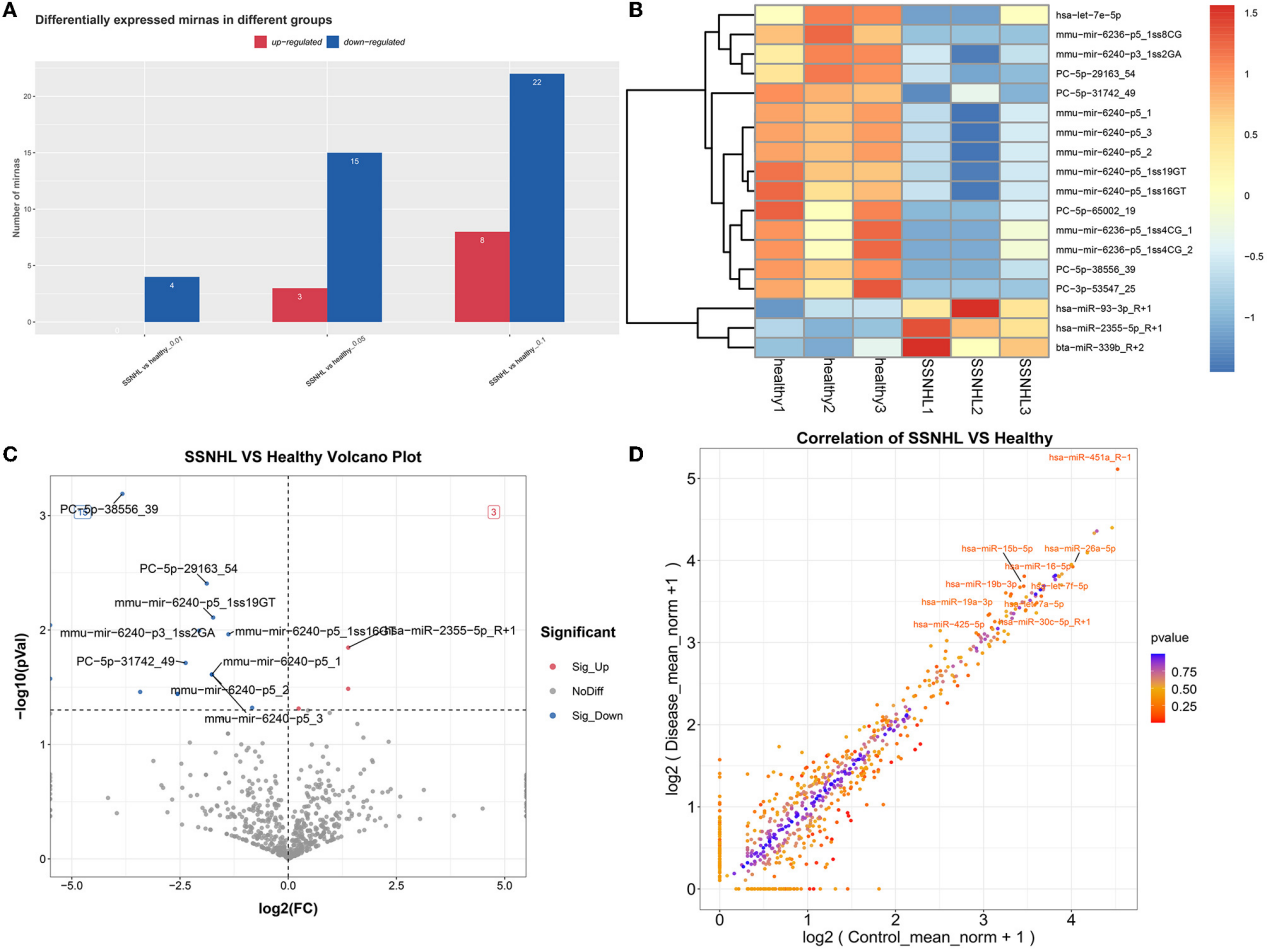
Screening of differentially expressed miRNAs (DE-miRNAs) in exosomes from SSNHL and healthy samples. Red: upregulated; blue: downregulated. **(A)** Statistical diagram of upregulated or downregulated miRNAs in the different groups. **(B)** Heat map of DE-miRNAs in exosomes from SSNHL and healthy samples. **(C)** Volcano map of DE-miRNAs from SSNHL and healthy samples. **(D)** Scatterplot of DE-miRNAs in SSNHL and healthy samples.

A total of 18 miRNAs were identified as DE-miRNAs in the SSNHL and healthy control samples based on the thresholds of |log_2_FC| > 1 and *P* < 0.05 (Figure 3A), which included PC-5p-38556_39, PC-5p-29163_54, mmu-mir-6240-p5_1ss19GT, mmu-mir-6236-p5_1ss8CG, mmu-mir-6240-p3_1ss2GA, mmu-mir-6240-p5_1ss16GT, hsa-miR-2355-5p_R+1, PC-5p-31742_49, mmu-mir-6240-p5_3, mmu-mir-6240-p5_2, mmu-mir-6240-p5_1, PC-3p-53547_25, hsa-miR-93-3p_R+1, PC-5p-65002_19, mmu-mir-6236-p5_1ss4CG_1, mmu-mir-6236-p5_1ss4CG_2, hsa-let-7e-5p, and bta-miR-339b_R+2. The identified DE-miRNAs also significantly differentiated SSNHL from the healthy control samples according to a heat map (Figure 3B). To identify miRNAs with the most significant differences, we generated a volcano map to observe the overall distribution of DE-miRNAs (Figure 3C) and a scatter diagram to visually depict differences in miRNA expression (Figure 3D).

### 3.4. Functional analyses

**Figure 4:**
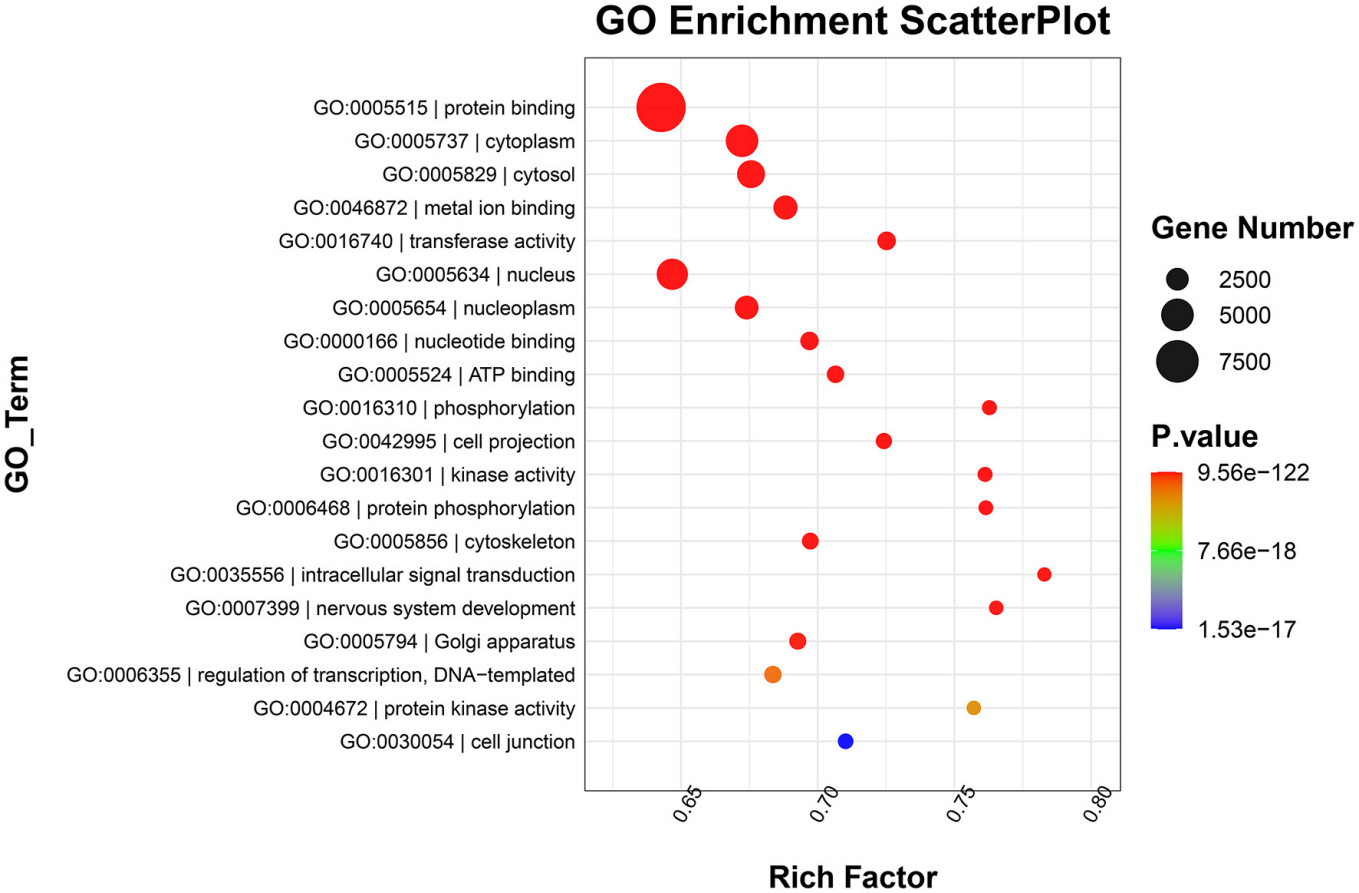
Gene Ontology (GO) enrichment scatterplot showing the top 20 terms with the lowest *p*-values. Rich factor: the proportion of the target gene number located in the GO to the total gene number located in the GO (rich factor = S gene number/B gene number).

**Figure 5:**
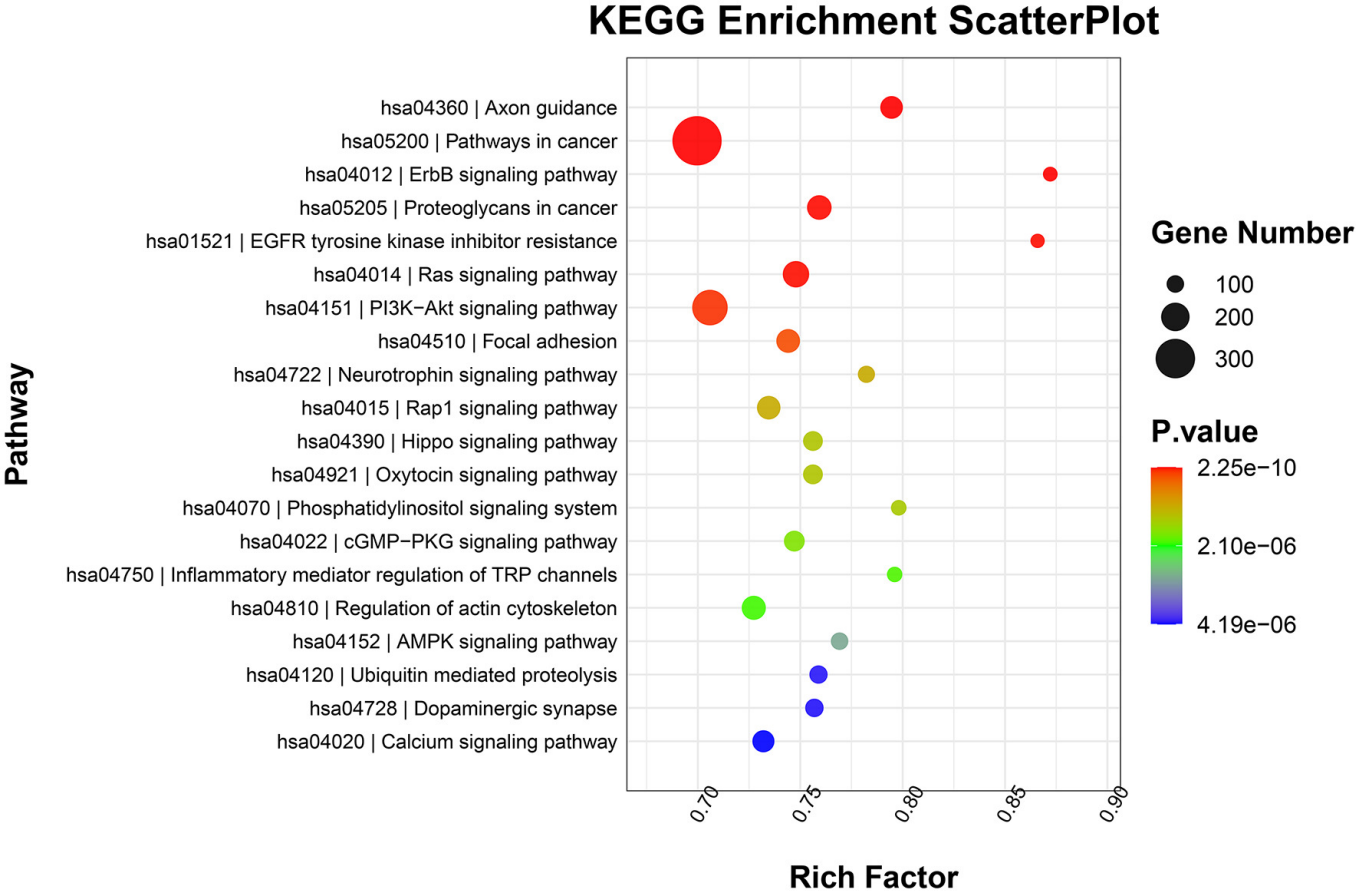
Kyoto Encyclopedia of Genes and Genomes (KEGG) pathway enrichment scatterplot showing the top 20 terms with the lowest *p*-values.

GO functional annotation analysis revealed that target genes of the identified DE-miRNAs in the top 20 terms were mainly related to “protein binding,” “metal ion binding,” “ATP binding,” and “intracellular signal transduction” (Figure 4). KEGG pathway enrichment analysis showed that the target genes of the identified DE-miRNAs were functionally enriched in the “Ras,” “Hippo,” “cGMP-PKG,” and “AMPK signaling pathways” (Figure 5).

### 3.5. Verification of sequencing by RT-qPCR

**Figure 6:**
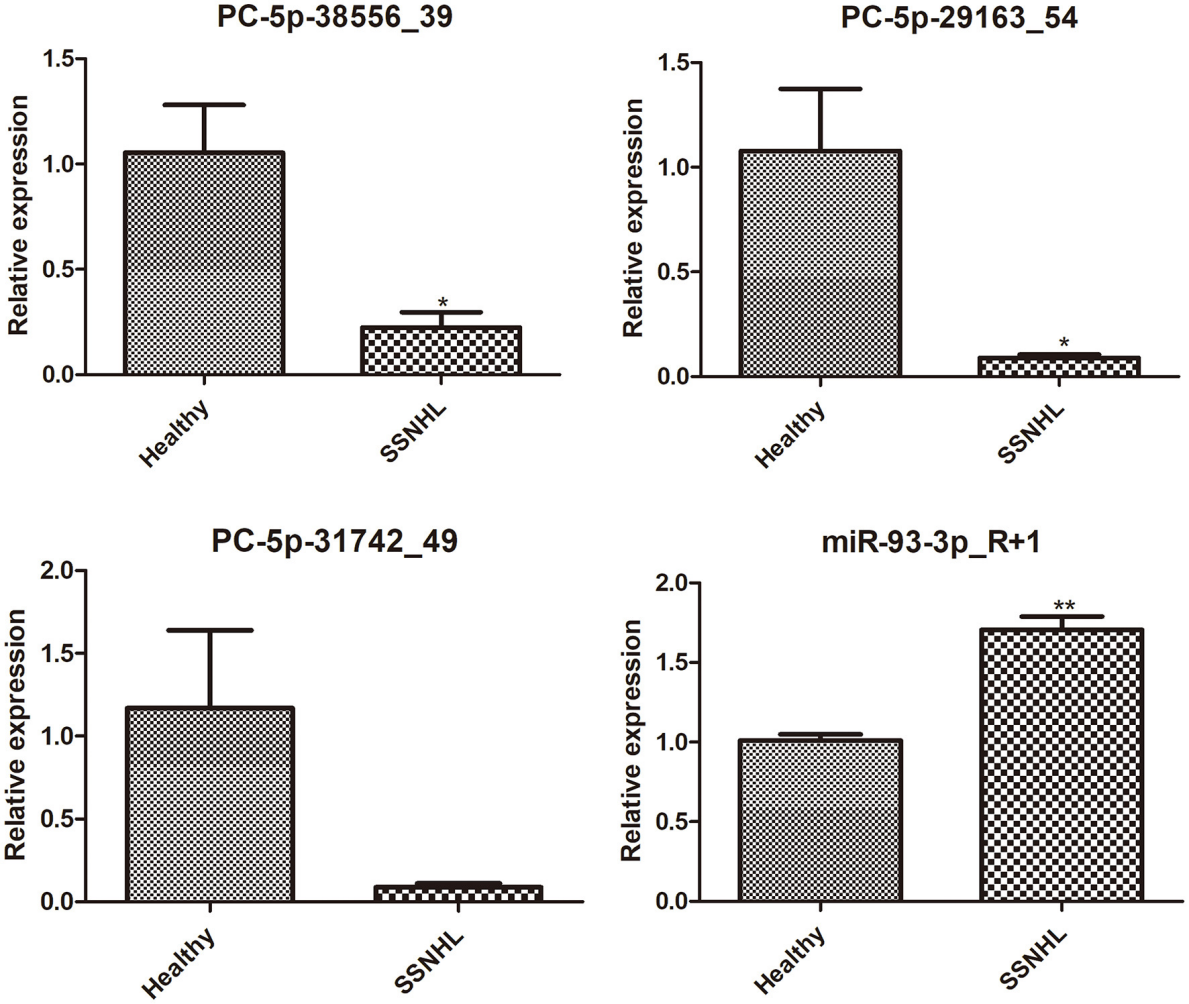
Expression Analysis of exosomal PC-5p-38556_39, PC-5p-29163_54, PC-5p-31742_49, and miR-93-3p_R+1 in exosomes isolated from healthy individuals and SSNHL patients (*n* = 3). *P < 0.05, **P < 0.01 vs. healthy controls.

Finally, four DE-miRNAs, including PC-5p-38556_39, PC-5p-29163_54, PC-5p-31742_49, and hsa-miR-93-3p_R+1, were chosen for RT-qPCR verification. It was found that compared with healthy controls, the expression levels of PC-5p-38556_39 and PC-5p-29163_54 were significantly downregulated (*P* < 0.05), whereas the expression level of miR-93-3p_R+1 was highly upregulated in exosomes from the SSNHL samples (*P* < 0.05, Figure 6). These results were consistent with the expression trends of the sequencing results. However, no significant difference was found in the level of PC-5p-31742_49 between exosomes from the SSNHL group and healthy samples (*P* > 0.05, Figure 6). All the results indicated that the consistency rate between sequencing and RT-qPCR was 75%, thereby indicating that the sequencing results were highly reliable.

## 4. Discussion

SSNHL is a common emergency in otolaryngology. Early recognition and treatment are crucial to improve hearing and alleviate tinnitus (32). Although pure tone audiometry results exhibit a variety of hearing curve types, systemic and intratympanic steroid therapy remains the main treatment for SSNHL (33). Owing to the lack of valuable early diagnostic markers, SSNHL can only be diagnosed after the onset of hearing loss through an audiological and medical history examination. Therefore, further studies surrounding potential SSNHL biomarkers are of great significance.

Scholars from various countries have studied SSNHL markers in plasma and serum, as well as from imaging perspectives. Elias et al. studied plasma malondialdehyde (MDA) activity in patients with SSNHL from the perspective of oxidative stress and studied the role of MDA in the prognosis of sudden deafness (34). Yao et al. studied inflammatory indexes in the peripheral blood of patients with SSNHL by using different audiogram shapes (35). Feng et al. investigated serum albumin and bone turnover biomarkers as potential prognostic markers for SSNHL (36, 37). Based on resting-state functional magnetic resonance imaging, Minosse et al. investigated the potential value of graph-theoretical measures as biomarkers for SSNHL (38). Liu et al. studied the potential value of regional homogeneity in the left cerebellum region as a neuroimaging biomarker for SSNHL (39). Fluctuations in exosome levels in the inner ear during disease states and their ability to carry and transmit intracellular signals have attracted increased interest (40). For instance, exosomes derived from inner ear stem cells increase the relative expression of miR-182-5p, alleviate gentamicin-induced ototoxicity, and improve the survival rate of HEI-OC1 cells (41), thus highlighting the potential use of exosomes as biomarkers for diseases of the inner ear. However, the inner ear is a complex structure located in the temporal bone, which makes it difficult to obtain cochlear specimens. Therefore, we collected peripheral venous blood for our study.

Cochlear ischemia-reperfusion injury is considered one of the crucial pathogeneses in SSNHL (42, 43). Hao et al. (44) found that exosomes derived from miR-21-transfected neural progenitor cells prevented hearing loss caused due to ischemia-reperfusion injury in mice by inhibiting inflammatory processes in the cochlea. Yang et al. (45) observed that cochlear spiral ganglion progenitor cell-derived exosomes reduced hearing loss caused due to ischemia-reperfusion injury in the cochlea by upregulating the expression of anti-inflammatory miRNAs (miR-21-5p, miR-26a-5p, and miR-181a-5p). In this study, we identified a total of eight personally sourced exosomal DE-miRNAs in SSNHL and compared them with controls, including PC-5p-38556_39, PC-5p-29163_54, hsa-miR-2335-5p_R+1, PC-5p-31742_49, PC-3p-53547_25, hsa-miR-93-3p_R+1, PC-5p-65002_19, and hsa-let-7e-5p. Moreover, miR-93 is an important regulatory factor in ischemia-reperfusion injury. The miR-93/IRAK4 (46) signaling pathway inhibits inflammation and cell apoptosis following cerebral ischemia-reperfusion injury. miR-93/STAT3 (47) and miR-93/PTEN (48) play protective roles in the inhibition of ischemia-reperfusion-induced liver injury and myocardial cell injury, respectively. miR-93 also plays a protective role in renal ischemia-reperfusion injury (49). Let-7e (50) is significantly altered in myocardial ischemia-reperfusion injury; Xu et al. (51) revealed that let-7e expression is reduced in noise-exposed rat cochlea, suggesting that *let-7e* and *fas* gene interactions are involved in noise-induced hearing loss. However, there are only a small number of studies on the relationship between exosomal DE-miRNAs and SSNHL.

The Hippo signaling pathway has been highly conserved throughout evolution (52). It is one of the most important signaling pathways that regulate the growth, differentiation, and regeneration of cochlear sensory and supporting cells (53). The regulation of the Hippo pathway can not only promote cell proliferation, hair cell regeneration, and neuronal reconnection (54) but also prevent aminoglycoside-induced cochlear injury/sensorineural deafness (55). However, its specific role in SSNHL requires further investigation.

Adenosine 5′-monophosphate activated protein kinase (AMPK) is a core regulator of cellular decomposition and anabolic pathways, which help maintain intracellular ATP levels (56). A decrease in AMPK levels reduces apoptosis and oxidative stress through the ROS-AMPK-bcl2 pathway in the cochlea and delays age-related hearing loss (57). Knocking out AMPK kinase in the cochlea can protect it from cisplatin or noise damage (58). We speculate that the AMPK signaling pathway may also play an important role in SSNHL.

Our study had certain limitations. First, we only verified the expression of some DE-miRNAs and did not verify the expression of all personally sourced DE-miRNAs. Second, the sample size was small. For instance, although PC-5p-31742_49 was identified as a DE-miRNA with downregulated expression, this result could not be confirmed using qRT-PCR in this study because of the large differences in the expression among samples; hence, further experiments with a larger sample size must be conducted to substantiate our findings. Finally, a machine learning model should be built to accurately verify whether the personally sourced DE-miRNAs can be used as SSNHL biomarkers; our future research will focus on the same.

## 5. Conclusion

To the best of our knowledge, this study is the first to establish DE-miRNA profiles of serum-derived exosomes in patients with SSNHL and conduct pathway analysis to determine the potential regulatory mechanisms involving exosomal miRNAs in SSNHL. The results of this study provide new ideas for further revealing the pathogenesis and potential biomarkers for SSNHL.

## Data availability statement

The original contributions presented in the study are publicly available. This data can be found here: National Center for Biotechnology Information (NCBI) BioProject, https://www.ncbi.nlm.nih.gov/bioproject/, PRJNA935061.

## Ethics statement

The studies involving human participants were reviewed and approved by the Medical Ethics Committee of Chongqing General Hospital (approval no. KYS2021-025-01). The patients/participants provided their written informed consent to participate in this study.

## Author contributions

JZ and WY: conception and design of the study and obtaining funding. JZ, HM, GY, and JK: acquisition of data. JZ, WS, LY, and SK: analysis and interpretation of data. HL: statistical analysis. JZ, HM, WS, and SK: drafting the manuscript. WY: revision of the manuscript for important intellectual content. All authors have read and approved the final version of the manuscript.
