# Supplementary material for: Differentially expressed miRNA profiles of serum-derived exosomes in patients with sudden sensorineural hearing loss
Source: Front Neurol. 2023 Jun 2;14:1177988. doi: 10.3389/fneur.2023.1177988 (PMC10273844; doi:10.3389/fneur.2023.1177988)
Supplement: Supplementary file 1 [file Data_Sheet_1.pdf]

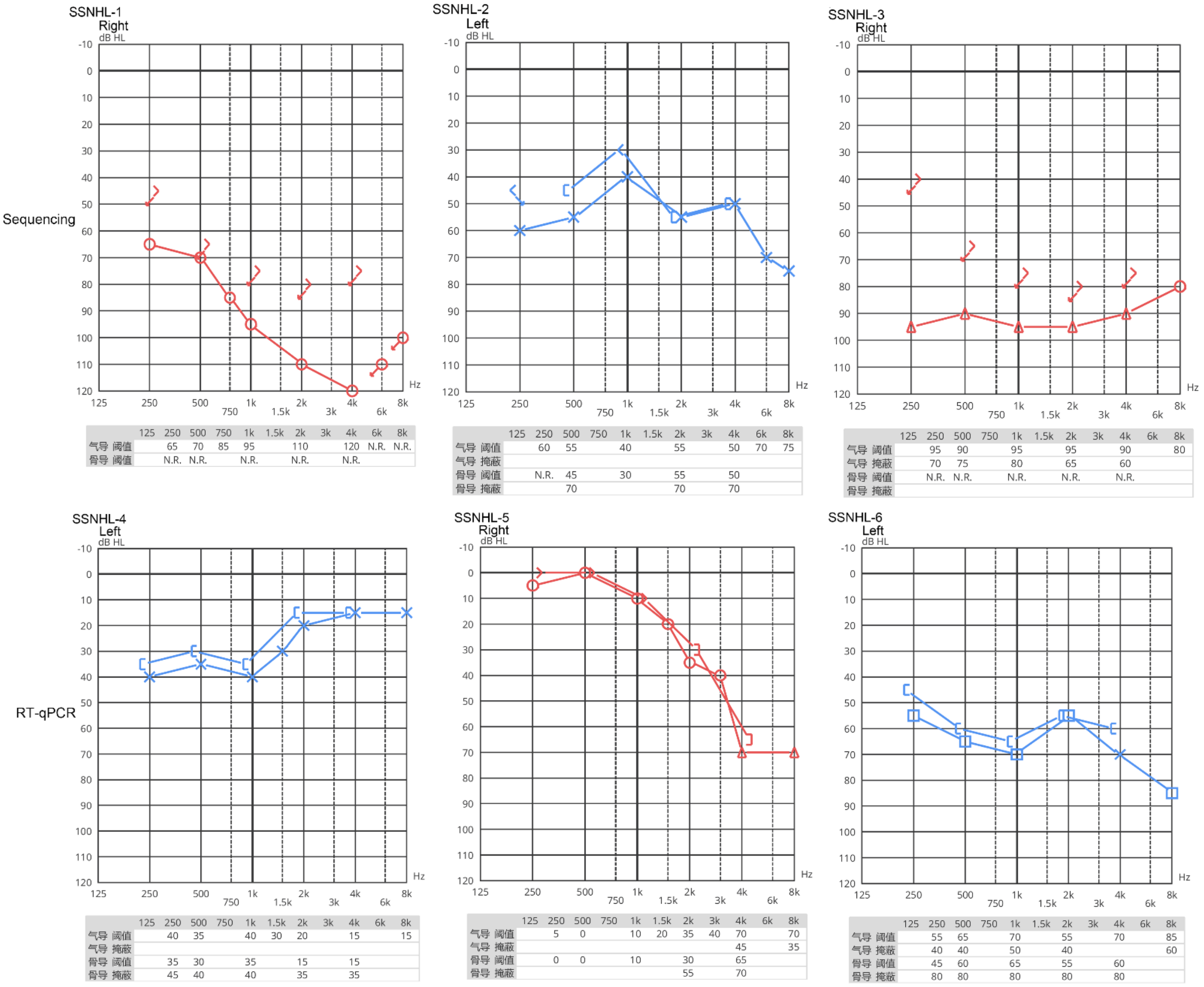

SSNHL-1 Right

	125	250	500	750	1k	1.5k	2k	3k	4k	6k	8k
气导 阈值		65	70	85	95		110		120	N.R.	N.R.
骨导 阈值		N.R.	N.R.		N.R.		N.R.		N.R.		

SSNHL-2 Left

	125	250	500	750	1k	1.5k	2k	3k	4k	6k	8k
气导 阈值		60	55		40		55		50	70	75
气导 掩蔽											
骨导 阈值		N.R.	45		30		55		50		
骨导 掩蔽			70				70		70		

SSNHL-3 Right

	125	250	500	750	1k	1.5k	2k	3k	4k	6k	8k
气导 阈值		95	90		95		95		90		80
气导 掩蔽		70	75		80		65		60		
骨导 阈值		N.R.	N.R.		N.R.		N.R.		N.R.		
骨导 掩蔽											

SSNHL-4 Left

	125	250	500	750	1k	1.5k	2k	3k	4k	6k	8k
气导 阈值		40	35		40	30	20		15		15
气导 掩蔽											
骨导 阈值		35	30		35		15		15		
骨导 掩蔽		45	40		40		35		35		

SSNHL-5 Right

	125	250	500	750	1k	1.5k	2k	3k	4k	6k	8k
气导 阈值		5	0		10	20	35	40	70		70
气导 掩蔽									45		35
骨导 阈值		0	0		10		30		65		
骨导 掩蔽							55		70		

SSNHL-6 Left

	125	250	500	750	1k	1.5k	2k	3k	4k	6k	8k
气导 阈值		55	65		70		55		70		85
气导 掩蔽		40	40		50		40				60
骨导 阈值		45	60		65		55		60		
骨导 掩蔽		80	80		80		80		80		

Supplementary figure 1 The audiograms of the sudden sensorineural hearing loss (SSNHL) cases (n = 6).

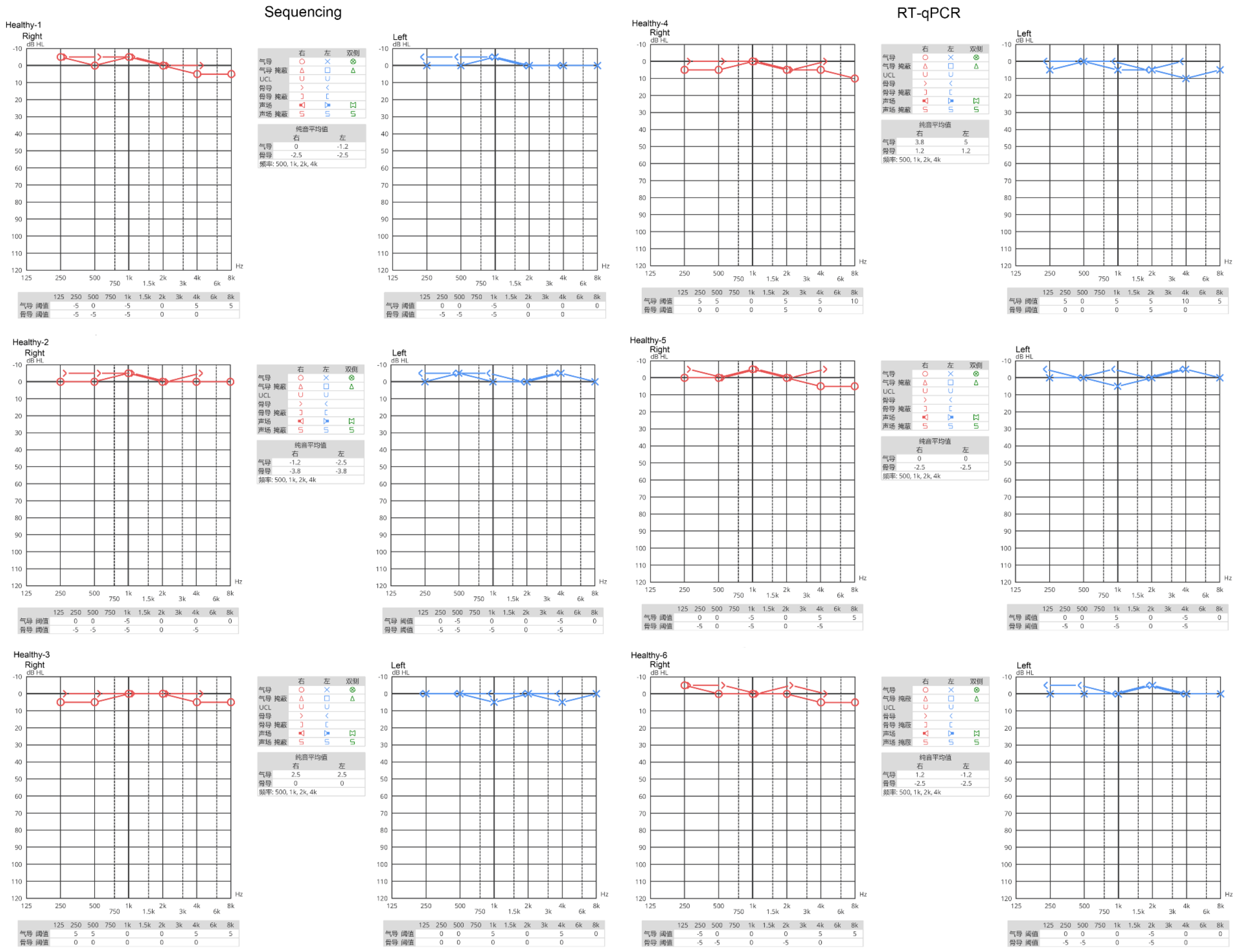

Supplementary figure 2 The audiograms of the healthy cases (n = 6).
